# Glucose metabolic brain patterns to discriminate amyotrophic lateral sclerosis from Parkinson plus syndromes

**DOI:** 10.1186/s13550-018-0458-5

**Published:** 2018-12-13

**Authors:** Martijn Devrome, Donatienne Van Weehaeghe, Joke De Vocht, Philip Van Damme, Koen Van Laere, Michel Koole

**Affiliations:** 1 Department of Nuclear Medicine and Molecular Imaging, Division of Nuclear Medicine, KU Leuven, Herestraat 49, 3000 Leuven, Belgium; 2 Department of Neurology, KU Leuven, Leuven, Belgium

**Keywords:** Positron emission tomography, ^18^F-FDG, Classification, Glucose metabolic brain patterns, Amyotrophic lateral sclerosis vs Parkinson plus

## Abstract

**Background:**

^18^F-FDG brain PET measures metabolic changes in neurodegenerative disorders and may discriminate between different diseases even at an early stage. The objective of this study was to classify patients with amyotrophic lateral sclerosis (ALS) and Parkinson plus syndromes (PP). To this end, different approaches were evaluated using generalized linear models and corresponding glucose metabolic brain patterns. Besides direct classification, healthy controls were also included to generate disease-specific metabolic brain patterns and to perform a classification using disease expression scores.

**Methods:**

ALS patients (*n* = 70) and PP patients (*n* = 33: 20 PSP, 3 CBD, and 10 MSA) were available from an existing database of patients with neuromuscular and movement disorders while age-matched healthy controls (*n* = 29) were selected from a prospective study. To generate both disease-discriminative (direct classification) and disease-specific (classification versus controls) metabolic brain patterns, data were spatially normalized and a principal component analysis (PCA) was performed prior to classification using either logistic regression (PCA-LR) or a support vector machine (PCA-SVM). Furthermore, a direct SVM approach was considered. To compare the three different approaches, Pearson correlations (*r*) between pattern expression scores and metabolic brain patterns were evaluated, while pairs of ALS- and PP-specific pattern expression scores were compared using the RV coefficient.

**Results:**

Classification between ALS and PP resulted in a sensitivity and specificity ≥ 0.82 for both direct classification and classification according to disease-specific pattern expression scores. PCA-LR, PCA-SVM, and SVM generated very similar metabolic brain patterns with voxelwise correlations ≥ 0.66, while all patterns allowed straightforward identification of ALS- and PP-specific brain regions of hyper- and hypometabolism. Moreover, pattern expression scores were highly correlated among different classifiers with a mean *r* of 0.94 while a RV coefficient ≥ 0.91 was found between pairs of ALS- and PP-specific pattern expression scores.

**Conclusion:**

We demonstrated that a classification between ALS and PP using expression scores of an ALS and PP metabolic brain pattern leads to a similar and high prediction accuracy as direct classification between ALS and PP. Classification performance and disease-specific metabolic patterns, which could support visual reading and improve insight in brain pathology, were very related for different classifiers.

## Background

^18^F-FDG brain positron emission tomography (PET) imaging is currently the quantitative imaging modality of choice for studying regional brain glucose metabolism and is increasingly being used for diagnostic and research purposes. Moreover, its clinical use has been established for specific diagnostic questions in neurodegenerative diseases [1, 2]. Over recent years, different methods have been developed to generate disease-specific metabolic patterns using ^18^F-FDG brain PET imaging. These brain patterns facilitate the differential diagnosis by providing a score representing the pattern expression of a specific brain disease. Moreover, these disease-specific glucose metabolic patterns allow the identification of specific brain networks, and therefore, a better understanding of the underlying mechanisms and topology of different brain disorders. For this purpose, a scaled subprofile model (SSM) [3] was used to identify brain networks in Alzheimer’s disease [4], Huntington’s disease [5], and Parkinson’s disease [6, 7], while corresponding methodological issues, such as network selection criteria and data log transform, were discussed by Spetsieris et al. [8]. Furthermore, a support vector machine (SVM) and independent component analysis (ICA) were considered to construct a spatial connectivity pattern in amyotrophic lateral sclerosis (ALS) [9]. SVM, originally proposed by Vapnik et al. [10], is based on the theory of structural risk minimization and is very efficient for high dimensional input data with only a few data sets available for training. Therefore, a SVM approach is particularly well suited for the analysis and classification of neuroimaging data in general and brain PET data in particular which was already demonstrated, e.g., for the classification of ALS patients and healthy controls using ^18^F-FDG PET [11], and for classification of healthy controls and patients with Alzheimer’s disease and mild cognitive impairment (MCI) using ^18^F-flutemetamol brain PET [12].

The aim of this study was to discriminate patients with amyotrophic lateral sclerosis (ALS) from Parkinson plus syndromes (PP). Discrimination between these two brain disorders is clinically relevant as motor neuron disease may occur in association with diverse parkinsonian manifestations [13–15]. Similarly, Parkinson’s disease can also manifest itself with atypical features, such as amyotrophy [16]. This overlap is further highlighted by the fact that both ALS and PP are considered neurodegenerative diseases with prion-like inclusions, i.e., TDP43 in ALS, Tau in corticobasal degeneration (CBD) and progressive supranuclear palsy (PSP) and alpha-synuclein in multiple system atrophy (MSA) [17]. In order to differentiate between ALS and PP, we used the framework of the generalized linear model (GLM). We demonstrated that different classifiers can be used to generate a disease-specific metabolic brain pattern if a data set of patients and healthy controls is available for training. Moreover, we aimed to evaluate whether classification based on expression scores of disease-specific metabolic brain patterns is more performant than a direct classification between two different brain disorders. The latter approach uses the same classifiers but does not require a training set of healthy controls and generates a disease-discriminative instead of a disease-specific brain pattern. For this study, we used a principal component analysis (PCA) prior to classification by logistic regression (LR) and SVM, and focused on the discrimination between patients with ALS and Parkinson plus syndromes (PP).

## Materials and methods

### Generalized linear model as framework for classification and disease-specific brain patterns

Consider a vectorized medical image *x* with corresponding discrete class label *t*. For a generalized linear model [18] with linear feature transform characterized by the transformation matrix *A*, the estimated output *y* is given by:

1 $$ y=f\ \left({w}^T Ax+b\right) $$

where *f* is the activation function. A training set of datasets {*x*_*n*_, *t*_*n*_} (*n* = 1, …, *N*) is used to determine the values of the weight vector *w* and constant *b* such that a linear decision boundary in feature space is created. However, since the input data space is high dimensional compared to the number of subjects (typically not exceeding a few hundreds), the classification problem is ill-conditioned. Therefore, the weight vector and constant are obtained from the training data by searching the minimum of a cost function which includes a loss function $\mathcal{L}\left(w,b\right)$ to fit the GLM to the data (Eq.1) and a regularization term $\mathcal{R}(w)$ to avoid overfitting [19]. As such, the weight vector is obtained by:

2 $$ {w}^{\ast }=\arg\ {\min}_w\ \left\{\frac{1}{N}{\sum}_{n=1}^N{\mathcal{L}}_n\left(w,b\right)+\lambda \mathcal{R}(w)\right\} $$

with *λ* the parameter determining the degree of regularization.

To differentiate between different neurodegenerative disorders, various (multiclass) classifiers can be applied which basically only differ in loss $\mathcal{L}\left(w,b\right)$ and regularization $\mathcal{R}(w)$. Moreover, these classifiers can be used to identify disease-specific brain patterns. The generation of these disease-specific patterns can be understood from the GLM framework (Eq.1), which can be rewritten as

3 $$ y=f\ \left(\left\langle {A}^Tw,x\right\rangle +b\right) $$

If the weight vector *w* is determined by training a classifier to discriminate between healthy controls and patients suffering from a specific brain disorder, (Eq.3) demonstrates that classification is based upon taking the inner product between *A*^*T*^*w* and *x*, thus projecting the image data x onto the vector *A*^*T*^*w* which could therefore be interpreted as an imaging biomarker or a disease-specific brain pattern.

#### PCA as feature transform

A principal component analysis (PCA) can be regarded as a dimensionality reduction technique by projecting the data onto a subspace spanned by the eigenvectors (principal components) of the data covariance matrix [20]. For this study, we used PCA to define the feature transformation matrix *A*, such that the rows of *A* are given by the principal components. Therefore, the marker *A*^*T*^*w* can be expressed as a weighted sum of principal components.

#### LR and SVM as classifiers

Logistic regression is a probabilistic discriminative model with an output variable *y* which can be interpreted as the probability that the transformed feature vector *Ax* belongs to a specific class. Within the GLM framework, LR corresponds to a logistic sigmoid as activation function (Eq.1) [21] with the logistic loss combined with elastic-net regularization (Eq.2) [22]. Since neighboring voxels are highly correlated, LR will only select a subset of representative voxels from the image data [23], generating less dense image patterns which are less appropriate for identifying disease-specific metabolic brain patterns or for assisting clinicians in visual readings. By applying PCA as a feature transform prior to LR classification, the corresponding pattern is given by a linear combination of voxelwise dense principal components, therefore retaining regional information of disease-related hyper- and hypometabolism.

On the other hand, SVM is an inherent binary classifier that defines a hyperplane between two classes by maximizing the margin representing the minimum distance from a separating hyperplane to the nearest data point. In case no hyperplane can be found separating the data completely, a soft margin SVM is used [24]. Within the GLM framework (primal formalism), a soft margin SVM is characterized by a step activation function (Eq.1) and hinge loss with L_2_-norm regularization (Eq.2). To avoid overfitting given the limited PET data and to ensure an interpretable pattern, a SVM with a linear kernel was considered [25]. Since SVM has an elegant dual formalism by expressing the classification model in terms of Lagrange multipliers [26], the weight vector is a sum of training data weighted by these Lagrange multipliers and can therefore already be interpreted as an image pattern. As such, a feature transformation prior to SVM classification is not mandatory. However, a SVM-only-based pattern is a superposition of noise and relevant metabolic brain information, and therefore, a feature transform such as PCA prior to SVM classification can still be useful as a denoising step to increase classification performance and improve pattern interpretability.

### Classification of ALS and PP patients using ^18^F-FDG brain PET imaging

#### Patient groups

An age- and gender-matched group of healthy controls (HC; *n* = 29, 62.4 ± 6.4 years; 16 M/13 F) was selected from a prospective study. Exclusion criteria were a history of neurological or psychiatric disorders, first-degree relatives of persons with dementia and psychiatric disorders needing treatment, major internal disorders (including diabetes), abnormalities on blood and urine screening, drug/alcohol abuse, abnormalities in clinical neurological examination or on a neuropsychological battery, or structural brain abnormalities on 3 Tesla MPRAGE T1 or FLAIR MRI sequences.

ALS patients (*n* = 70, 62.1 ± 12.5 years; 44 M/26 F) (*9*) and PP patients (*n* = 33, 68.5 ± 7.9 years; 21 M/12 F; preferential diagnosis: 20 PSP, 3 CBD, and 10 MSA) were selected from an existing database of referrals made to the tertiary neuromuscular and movement disorder clinic at the University Hospital Leuven, Belgium. None of the patients had a history of other neurological disorders. ALS patients were diagnosed using the Awaji criteria [27]. All ALS patients underwent full neurological evaluation and electrodiagnostic testing as part of their clinical workup by an experienced specialist in neuromuscular disorders. None of the patients showed evidence of respiratory compromise or nutritional abnormalities, such as dehydration or ketosis, at the time of the ^18^F-FDG-PET scan.

#### ^18^F-FDG brain PET data acquisition, reconstruction, and preprocessing

All subjects fasted at least 6 h before the ^18^F-FDG PET acquisition. Before ^18^F-FDG injection, blood glucose was measured to ensure a glucose level< 160 mg/dl. ^18^F-FDG (153.5 ± 15.0 MBq) was injected intravenously under standard conditions, that is, subjects lying supine in a dimly lit, quiet room, with ears and eyes open. Thirty minutes after ^18^F-FDG injection, a static scan of 30 min was acquired. ^18^F-FDG PET scans were acquired using ECAT HR+ camera for the ALS patients, HC and 19 PP patients (Siemens, Erlangen, Germany) and a Biograph 16 HiRez PET/CT camera for 20 PP patients. On the HR+ camera, attenuation-corrected ^18^F-FDG images were reconstructed using a 68Ge/68Ga transmission scan, resulting in full width at half maximum (FWHM) of 7 mm. On the HiRez PET/CT camera, ^18^F-FDG images were reconstructed using iterative ordered-subset expectation maximization (OSEM) with four iterations over four subsets (FWHM 8 mm). During the acquisition, the subject’s head was immobilized by means of a vacuum pillow. Reconstructed PET data were normalized to Montreal Neurological Institute (MNI) space using a brain ^18^F-FDG template using (PMOD version 3.7, PMOD Inc., Zürich, Switzerland). Subsequently, scans were smoothed by applying a three-dimensional isotropic 6 mm Gaussian kernel, after which the images were masked (full brain mask) and vectorized. In order to correct for intersubject variability including potential scanner dependencies, the vectorized images were normalized (divided by *L*_2_-norm) after performing a demean, i.e., mean value for every subject was subtracted.

#### Training and validation

**Fig. 1:**
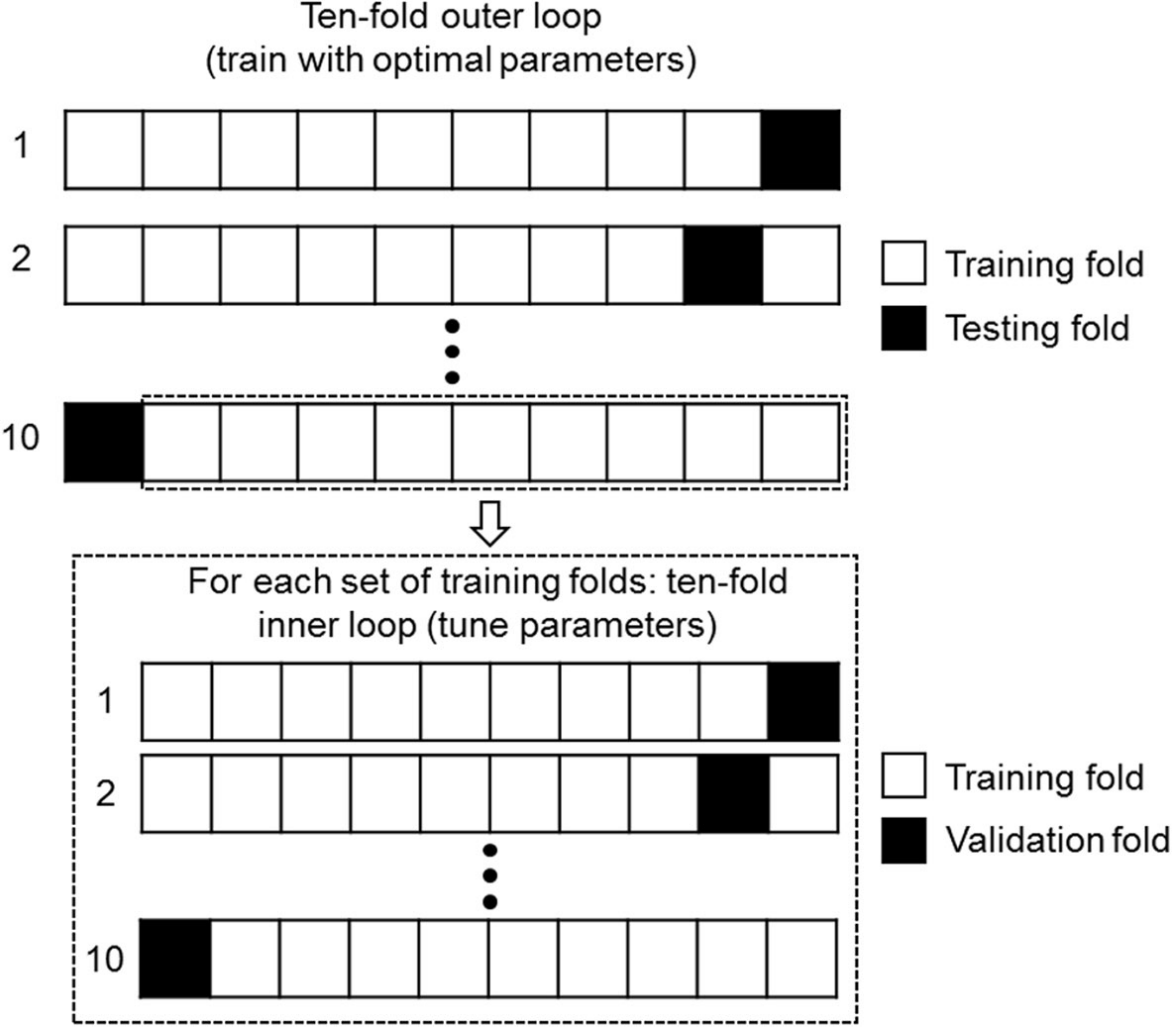
Schematic overview of the tenfold cross-validation. Within each tenfold of the outer loop for training and testing, a tenfold cross-validation inner loop was defined over the training folds to optimize classifier-specific parameters

SVM and LR, combined with a PCA prior to classification (PCA-SVM and PCA-LR), were evaluated to classify ALS and PP patients and to generate ALS- and PP-specific metabolic brain patterns. In addition, SVM without prior feature transform was evaluated. For the SVM implementation, a *L*_1_ soft margin SVM with linear kernel was considered (MATLAB version 2016b, The MathWorks Inc., Massachusetts, USA). As illustrated in Fig. 1, all binary classifiers were trained and tested by applying tenfold cross-validation (CV) while preserving the balance in size of both classification groups. Within each tenfold, an inner loop tenfold CV was defined over the training folds to tune classifier-specific parameters. For SVM, the soft margin and kernel scale hyperparameters were optimized by applying a grid search over the nested inner loop while for LR, the parameters controlling the elastic-net and overall regularization were determined by cyclical coordinate descent using the Glmnet package [28]. The tenfold CV scheme was iterated ten times randomly, and therefore, performance results were interpreted as average values together with the corresponding standard deviation. Classification performance was assessed by the receiver operating characteristic (ROC) curves with corresponding area under the curve (AUC) values, and by the sensitivity and specificity (true positives are the patients having ALS diagnosed by the model as ALS). Moreover, Pearson correlations were determined between the individual pattern expression scores (averaged over the ten random iterations) as determined by PCA-SVM, PCA-LR, and SVM to compare the output of three classifiers for direct classification between ALS and PP. Besides direct classification between ALS and PP, ALS- and PP-specific brain patterns were generated by classifying HC versus ALS and PP respectively using either PCA-SVM, PCA-LR, or SVM. By applying tenfold CV over ten random validation schemes, a series of ten pairs of pattern expression scores for both the ALS- and PP-specific brain pattern was obtained for each subject. To evaluate a classification based on these ALS- and PP-specific pattern expression scores, a linear SVM was trained and evaluated by tenfold CV and ROC curves with corresponding AUC, sensitivity, and specificity were determined to allow comparison with direct classification between ALS and PP. Additionally, the RV coefficient, which is a multivariate generalization of the squared Pearson correlation coefficient with values in the interval [0, 1] [29], was determined for the pairs of individual ALS- and PP-specific pattern expression scores (averaged over the ten random iterations) as determined by the three classifiers to compare the output of PCA-SVM, PCA-LR, and SVM in terms of disease-specific pattern expression scores.

To compare the glucose metabolic brain patterns obtained with SVM, PCA-SVM, and PCA-LR, all image data were included to train the three classifiers for both classification between ALS and PP and classification between HC and either ALS or PP. Resulting discriminative and disease-specific metabolic brain patterns were compared using a voxelwise Pearson correlation coefficient.

## Results

### Direct classification between ALS and PP

**Fig. 2:**
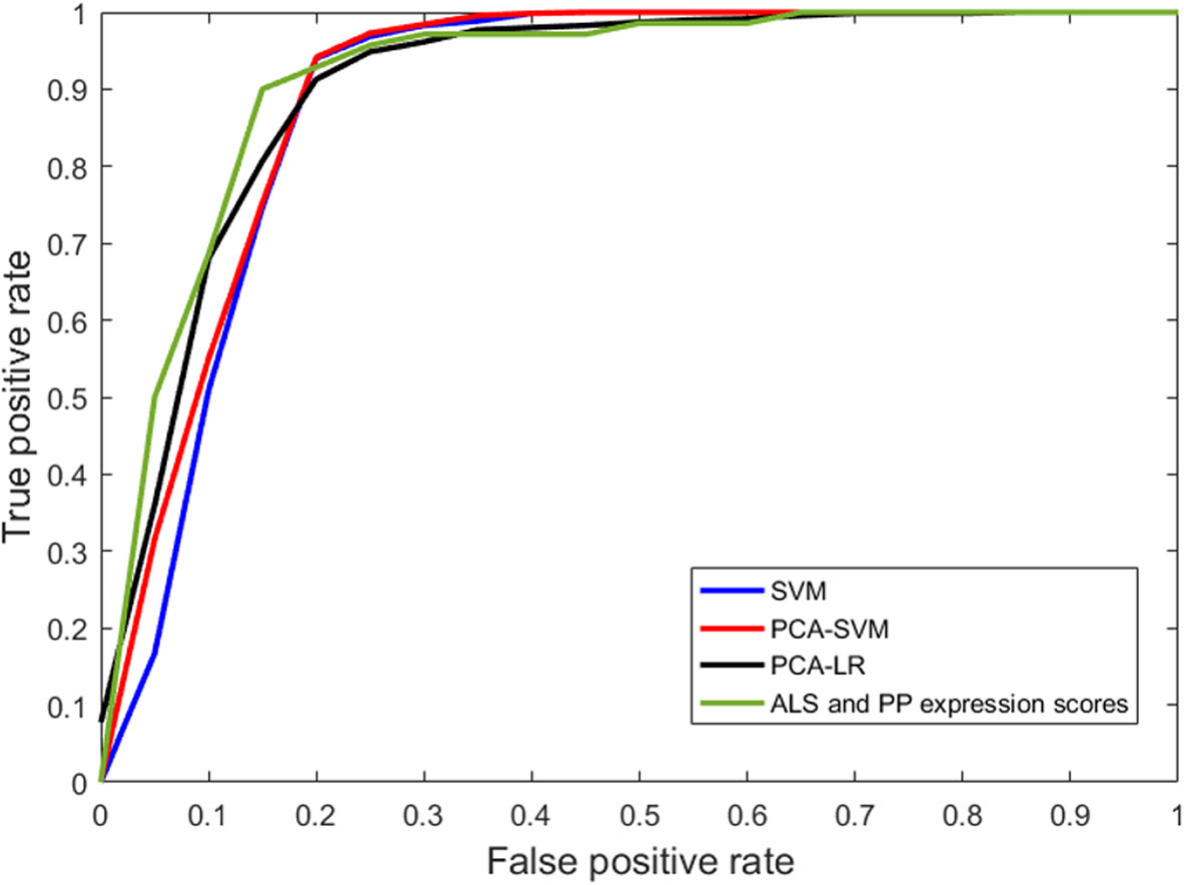
Average ROC curves for the classification between ALS and PP using SVM, PCA-SVM, and PCA-LR respectively combined with the ROC curve for a SVM classification between ALS and PP based on the ALS- and PP-specific pattern expression scores as determined by PCA-SVM using a healthy control group and projecting individual ^18^F-FDG PET scans onto the ALS- and PP-specific metabolic brain patterns

**Fig. 3:**
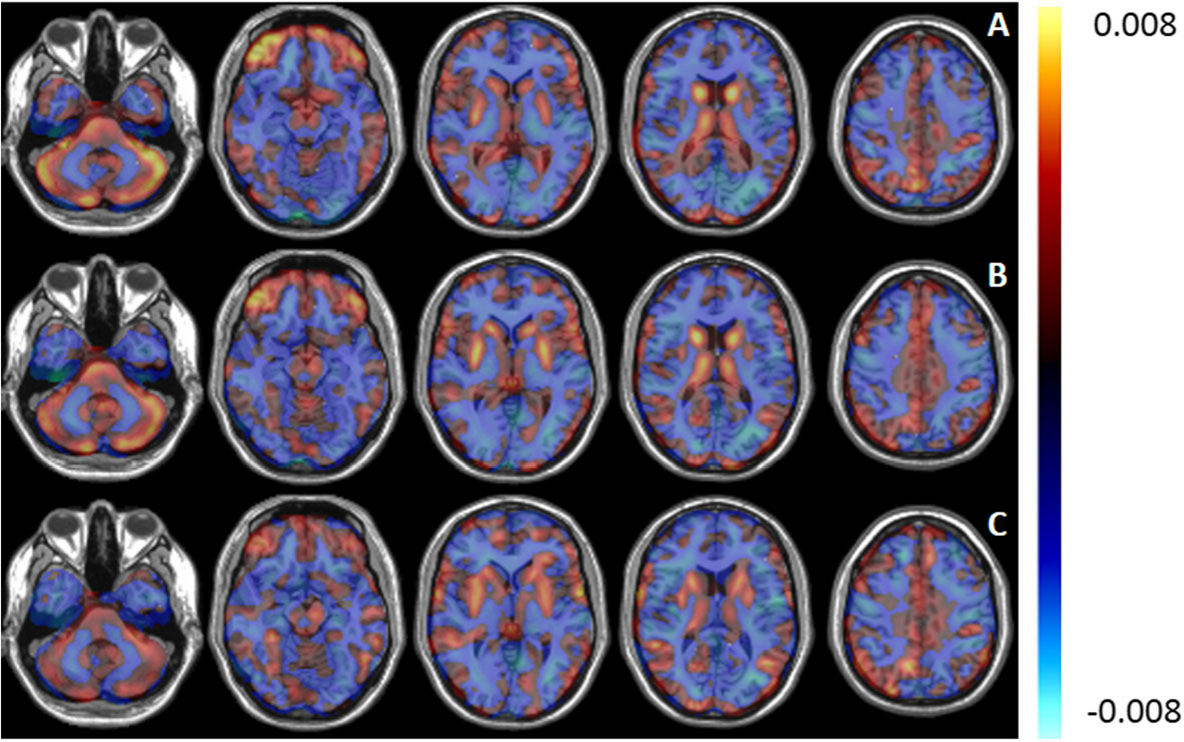
Discriminative patterns (arbitrary units) between ALS and PP determined by PCA-LR (**a**), PCA-SVM (**b**), and SVM (**c**). The values in each voxel represent the weights of the contribution of the voxel for the classifier. Positive values (red) indicate regions of relative hypermetabolism of ALS patients compared to PP patients, whereas negative values (blue) correspond to relative hypometabolism of ALS patients compared to PP patients

The average ROC curves obtained by SVM, PCA-SVM, and PCA-LR for direct classification between ALS and PP are given in Fig. 2. The corresponding AUC values are given in Table 1 together with the sensitivity and specificity. Furthermore, the Pearson correlation coefficient (*r*) between the individual pattern expression scores, as determined by the three classifier, is given in Table 3. For each classifier, the 3D discriminative pattern *A*^*T*^*w* (see Eq. 2), obtained by using all image data for training, is illustrated in Fig. 3. The values in each voxel are the weights of the contribution of the classifier. The positive values, marked in red, indicate regions of relative hypermetabolism in ALS patients compared to PP patients, whereas the negative values, indicated in blue, correspond to relative, regional hypometabolism in ALS patients compared to PP patients.

**Table 1:** Performance measures for classification between ALS and PP for SVM, PCA-SVM, and PCA-LR: sensitivity, specificity, and AUC. In addition, the accuracy measures are also given for a SVM classification between ALS and PP based on the ALS- and PP-specific pattern expression scores as determined by PCA-SVM by using a healthy control group and projecting individual ^18^F-FDG PET scans onto the ALS and PP-specific metabolic brain patterns

	Sensitivity	Specificity	AUC
SVM	0.86 ± 0.01	0.82 ± 0.01	0.89 ± 0.01
PCA-SVM	0.86 ± 0.02	0.82 ± 0.02	0.90 ± 0.01
PCA-LR	0.85 ± 0.02	0.82 ± 0.02	0.90 ± 0.01
Classification based on ALS and PP expression scores (SVM)	0.90 ± 0.02	0.83 ± 0.01	0.91 ± 0.01

### Classification between ALS and PP using disease-specific pattern expression scores

**Fig. 4:**
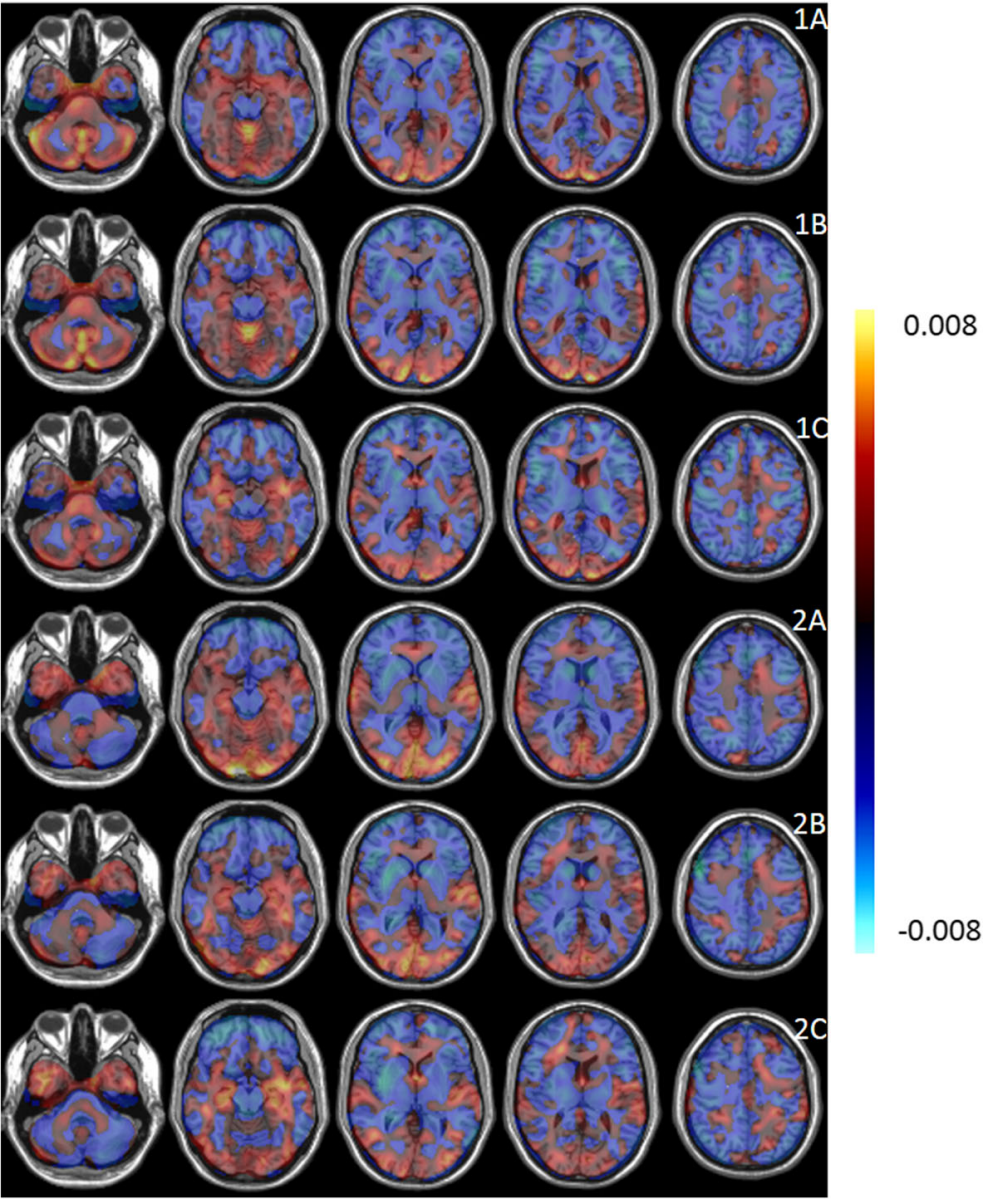
Disease-specific patterns (arbitrary units) for ALS (1) and PP (2) determined by PCA-LR (**a**), PCA-SVM (**b**), and SVM (**c**). The values in each voxel represent the weights of the contribution of the voxel for the classifier. Positive values (red) indicate regions of hypermetabolism of ALS or PP patients compared to HC whereas negative values (blue) correspond to hypometabolism of ALS or PP patients compared to HC

**Fig. 5:**
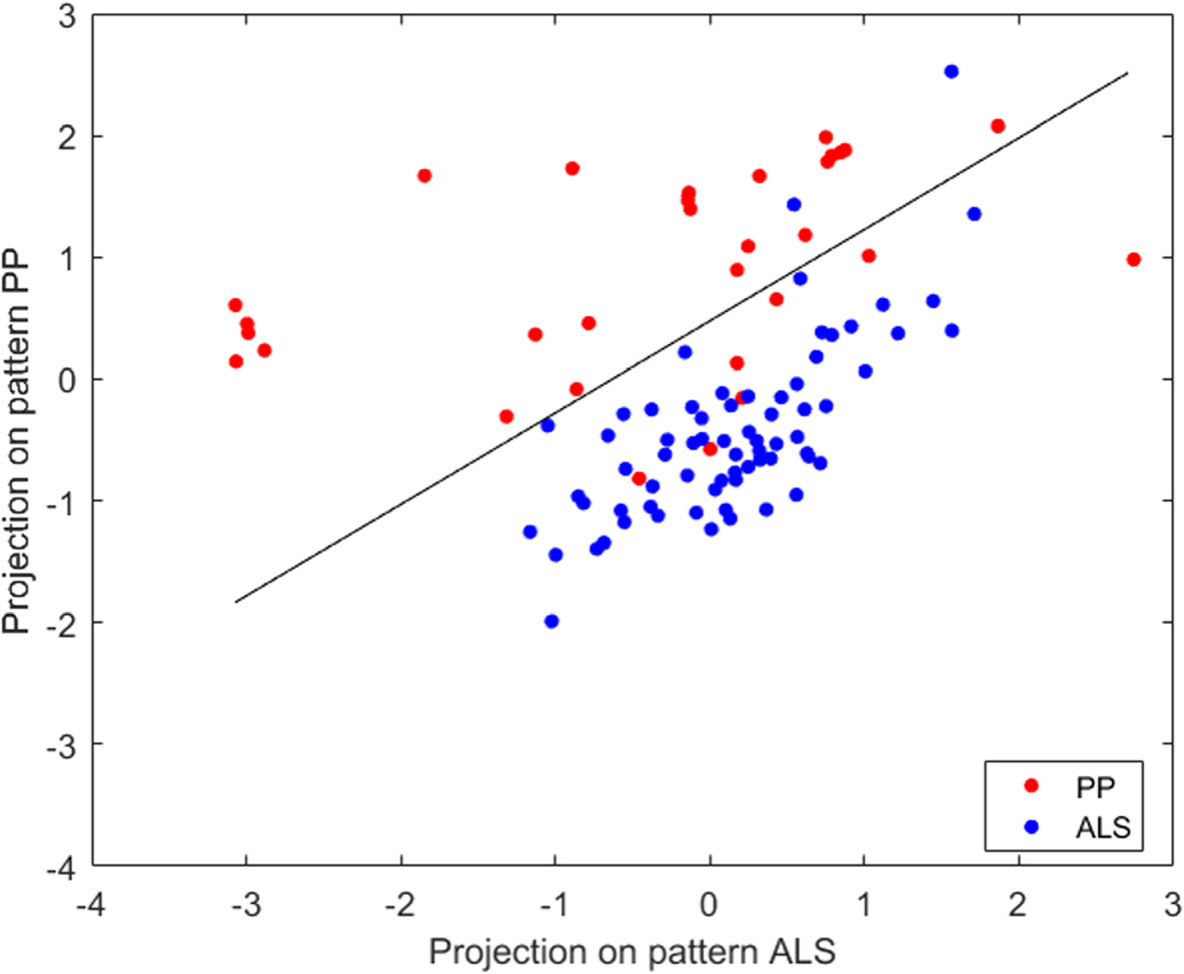
Individual ALS- and PP-specific pattern expression scores by projecting the corresponding ^18^F-FDG PET scan onto a ALS- and PP-specific metabolic brain pattern as determined by PCA-SVM. A linear SVM is used classify both groups based on these disease expression scores

The performance measures for a classification between ALS versus HC and PP versus HC for SVM, PCA-SVM, and PCA-LR are given in Table 2. RV coefficients between the pairs of individual ALS- and PP-specific pattern expressions scores are given in Table 3 for the three classifiers. Since the RV coefficients demonstrated a high correlation between ALS- and PP-specific pattern expression scores for all three classifiers, only the individual ALS- and PP-specific pattern expression scores as determined by PCA-SVM were used to classify both groups using a linear SVM. The average ROC curve for this SVM classification, based on disease-specific pattern expression scores and evaluated by a tenfold CV, is illustrated in Fig. 2, with a corresponding AUC of 0.91 and a prediction accuracy of 85% (point along ROC curve with identical sensitivity and specificity). Individual ALS- and PP-specific pattern expression scores, averaged over the ten random iterations, as determined by PCA-SVM are plotted in Fig. 5, together with the hyperplane (in this case a line) separating both groups as determined by a linear SVM. ALS- and PP-specific metabolic brain patterns, obtained by differentiating ALS and PP patients from HC and including all image data for training, are illustrated in Fig. 4 for the three classifiers. Again positive values, marked in red, indicate regions of relative hypermetabolism in ALS or PP patients compared to HC, whereas the negative values, indicated in blue, correspond to relative, regional hypometabolism in ALS or PP patients compared to HC. Specifically for the ALS-specific brain pattern, relative hypometabolism in the frontal and parietal cortex and relative hypermetabolism in the anteromedial temporal cortex was observed while the PP-specific brain patterns demonstrated relative hypometabolism in the striatum, pons and frontal cortex, and cerebellum. The voxelwise Pearson correlation coefficients between the disease-specific patterns as determined by the three classifiers are given in Table 3, demonstrating a consistently high correlation (≥ 0.66) between disease-specific glucose metabolic brain patterns.

**Table 2:** Performance measures for classification between ALS versus HC and PP versus HC for SVM, PCA-SVM, and PCA-LR: sensitivity, specificity, and AUC

	Sensitivity	Specificity	AUC
ALS			
SVM	0.98 ± 0.01	0.97 ± 0.01	0.99 ± 0.01
PCA-SVM	0.98 ± 0.01	0.99 ± 0.01	0.99 ± 0.01
PCA-LR	0.97 ± 0.02	0.90 ± 0.02	0.97 ± 0.01
PP			
SVM	0.98 ± 0.01	0.97 ± 0.01	0.99 ± 0.01
PCA-SVM	0.92 ± 0.01	0.94 ± 0.01	0.99 ± 0.01
PCA-LR	0.94 ± 0.02	0.97 ± 0.02	0.98 ± 0.01

**Table 3:** RV coefficient between pairs of individual ALS- and PP-specific pattern expression scores (averaged over ten random iterations) as determined by SVM, PCA-SVM, and PCA-LR by projecting corresponding ^18^F-FDG PET scans on a ALS- and PP-specific pattern respectively, combined with the Pearson correlation coefficient (*r*) between the individual projection scores, again averaged over ten random iterations, onto a discriminative brain pattern for ALS and PP (direct classification) for SVM, PCA-SVM, and PCA-LR. The voxelwise correlation coefficients between the ALS and PP metabolic brain patterns, determined by the different classifiers are also reported

		RV coefficient for disease expression scores	*r* for prediction scores ALS vs PP	*r* ALS pattern	*r* PP pattern
SVM	PCA-SVM	0.94	0.98	0.89	0.79
SVM	PCA-LR	0.91	0.92	0.70	0.66
PCA-SVM	PCA-LR	0.95	0.92	0.86	0.85

## Discussion

The aim of this study was twofold. First, we demonstrated that a direct classification between different brain disorders such as ALS and PP is equally performant as a classification based on the individual expression scores of disease-specific glucose metabolic patterns. More specifically for discriminating between ALS and PP, a linear SVM using ALS- and PP-specific pattern expression scores classified the two groups with a AUC of 0.91 (as determined by PCA-SVM and illustrated in Fig. 5), which is very close to the AUC of 0.90 for a direct classification by PCA-SVM (Table 1)*.* Moreover, the high classification accuracies allow both approaches to support the sometimes difficult early differential diagnosis between ALS and PP in a clinical setting. This is particularly relevant if the main clinical question is to discriminate between two specific diseases as it obviates the need for healthy controls to generate disease-specific glucose metabolic patterns. In terms of direct classification between ALS and PP, PCA-LR, SVM, and PCA-SVM performed very similar (see ROC curves in Fig. 2 and Table 1), wherein additionally, the disease-specific pattern expression scores between these three different classifiers also strongly correlated (Table 3). Therefore, no specific approach could be put forward as the classifier of choice using either a direct approach or by including a group of healthy controls. Importantly, these findings confirmed that SVM is a suitable classifier for ^18^F-FDG brain PET imaging.

Second, we showed that different classifiers can be used to generate both disease-discriminative (Fig. 3) and disease-specific (Fig. 4) glucose metabolic brain patterns using ^18^F-FDG brain PET imaging. Since ALS patients match a positive and PP patients a negative pattern score, positive weights in the disease discriminative pattern constitute hypermetabolism in ALS patients compared to PP patients while negative pattern weights correspond to hypometabolic brain regions in ALS patients compared to PP patients. This same holds for the disease-specific ALS and PP pattern where an ALS or PP patient corresponds to a positive pattern score and thus positive pattern weights correspond to hypermetabolism in ALS and PP patients compared to healthy controls while negative weights represent hypometabolic brain regions in ALS and PP patients compared to healthy controls. Correlating the pairs of individual projection scores for a ALS- and PP-specific metabolic brain pattern determined by each of the three methods resulted in a RV coefficient of 0.93 on average (Table 3). Similarly, the voxelwise Pearson correlation coefficient for the disease-specific patterns demonstrated the strong correlation between ALS and PP patterns as determined by the three classifiers, with a lower correlation between patterns determined by SVM and PCA-LR (*r* value of 0.70 for ALS and 0.66 for PP respectively). The lower correlation between SVM and PCA-LR patterns could be explained by the fact that SVM and PCA handle noise and intersubject variability differently. Although, these patterns demonstrated visually the same main regions of hyper- and hypometabolism. Overall, the generated ALS- and PP-specific patterns were very similar to the ALS and PP patterns that have been discussed extensively in literature. Specifically, for ALS patients, relative hypometabolism in the frontal and parietal cortex and relative hypermetabolism in the anteromedial temporal cortex, in the absence of concomitant frontotemporal dementia, cerebellum, and brainstem have been observed [11]. In contrast, PP patients have relative hypometabolism in the striatum and according to the subtype, relative hypometabolism in the pons and frontal cortex (PSP), relative hypometabolism in the cerebellum (MSA), and a very asymmetric relative hypometabolic pattern in CBD [11, 30–32]. As such, the generated PP pattern is a composition of different subtype patterns and therefore masks some subtype-specific characteristics such as the CBD-specific hypometabolic asymmetry. On the other hand, the highly accurate classification results implied that the generated patterns are to be considered as disease-specific for ALS and PP or discriminative between ALS and PP. Therefore, the glucose metabolic brain patterns reflect disease pathogenesis and hold information about diagnosis and prognosis. This way, this pattern not only supports differential diagnosis but can also have an added value for assisting visual reading.

While the SVM approach is in line with previous SVM implementations that were used for the classification of ALS patients [11], the PCA-LR classifier closely resembles the SSM/PCA approach proposed by Eidelberg et al. [8]. The SSM/PCA has often been applied to identify metabolic disease-specific patterns and corresponding network expression scores, such as to generate a Parkinson’s disease-related pattern based on metabolic PET data [7]. However, the SSM/PCA approach includes several preprocessing steps other than PCA such as double demean and log transform, and selects the relevant principal components for the disease-specific glucose metabolic brain pattern in a more heuristic way [8]. On the other hand, our approach assigned a proper weight for every single principle component during the training step of the classifier. This way, all principle components were taken into account. For a SVM classification, a combination with PCA did not improved the prediction accuracy of a classification between ALS and PP (Table 2). Nevertheless, a feature transform prior to SVM classification can still be beneficial to reduce the impact of noise on classification performance and to improve the interpretability of disease-related glucose metabolic brain patterns (Fig. 5). For this study, a PCA was applied as feature transform since it has proven its usefulness for PET imaging [7]. However, other feature transforms such as factor analysis may be applied prior to classification. Furthermore, interpretable weight maps and patterns could be created by including spatial regularization such as total variation (TV) to couple neighboring voxels and to recover the predictive disease-related brain regions more accurately [33, 34]. However, TV optimization tends to be slow and challenging since all voxels are spatially coupled.

Some limitation to this study is to be considered. First of all, PET images were normalized to MNI space using PET-only information since magnetic resonance (MR) images were not available. Although this approach could reduce the accuracy of the normalization, the impact on the resulting discriminative and disease-specific patterns is expected to be limited. Moreover, since high-resolution anatomical MR data were not available, we did not consider a patient-specific gray matter mask to spatially constrain the disease-specific or discriminative brain patterns nor did we apply prior knowledge to generate these pattern. These approaches could prove beneficial and will be considered in the future. Another limitation is that not all PET data were acquired with the same PET scanner. However, potential scanner differences were accounted for during the preprocessing steps which comprised a histogram normalization and demean. Moreover, it has been shown that resolution properties of the two PET scanners that have been used for this study were very similar for the applied reconstruction settings [35].

In terms of patient groups, this study was limited to ALS and PP patients. We did not extend the PP group to a more general ALS mimics group, since the main focus of this study was to evaluate different approaches using ^18^F-FDG brain PET only from a methodological point of view. Finally, we did not include patients with Idiopathic Parkinson’s disease (iPD) in the group of patients with Parkinsonian symptoms. Since ^18^F-FDG brain PET is not part of the routine clinical workup, in contrast to imaging of the dopamine transporter (DAT), ^18^F-FDG brain PET data are limited for this patient group. Nevertheless, efforts are being made to extend the ^18^F-FDG brain PET data with iPD patients for future classification and generation of disease-specific or discriminative glucose metabolic brain patterns for ALS, iPD, and different subtypes of PP.

## Conclusion

We demonstrated that the overall prediction accuracy for classifying neurodegenerative disorders such as ALS and PP using the expression scores of disease-specific metabolic brain patterns was very high (> 80%) and equaled the performance of a direct classification approach. Although disease-specific glucose metabolic patterns require the inclusion of healthy controls, these patterns provide disease-related relative hyper- and hypometabolic information, and therefore, can support visual reading. We also showed that the performance of three different classifiers was equivalent with very similar glucose metabolic brain patterns. These findings confirm SVM as an appropriate classifier for ^18^F-FDG brain PET imaging and for generating disease-specific metabolic brain patterns.

## Abbreviations

ALS: Amyotrophic lateral sclerosis
AUC: Area under the curve
CBD: Corticobasal degeneration
CV: Cross validation
DAT: Dopamine transporter
FDG: Fluorodeoxyglucose
FN: False negatives
FP: False positives
FWHM: Full width at half maximum
GLM: Generalized linear model
HC: Healthy controls
iPD: Idiopathic Parkinson’s disease
LR: Logistic regression
MNI: Montreal Neurological Institute
MR: Magnetic resonance
MSA: Multiple system atrophy
OSEM: Ordered-subset expectation maximization
PCA: Principal component analysis
PET: Positron emission tomography
PP: Parkinson plus
PSP: Progressive supranuclear palsy
ROC: Receiver operating characteristics
SSM: Scaled subprofile model
SVM: Support vector machine
TP: True positives
TV: Total variation

## References

[1] Marcus C, Mena E, Subramaniam RM (2014). Brain PET in the diagnosis of Alzheimer's disease. Clin Nucl Med.

[2] Silverman DH (2004). Brain 18F-FDG PET in the diagnosis of neurodegenerative dementias: comparison with perfusion SPECT and with clinical evaluations lacking nuclear imaging. J Nucl Med.

[3] Moeller JR, Strother SC, Sidtis JJ, Rottenberg DA (1987). Scaled subprofile model: a statistical approach to the analysis of functional patterns in positron emission tomographic data. J Cereb Blood Flow Metab.

[4] Scarmeas N, Habeck C, Anderson KE, Hilton J, Devanand DP, Pelton GH (2004). Altered PET functional brain responses in cognitively intact elderly persons at risk for Alzheimer disease (carriers of the epsilon4 allele). Am J Geriatr Psychiatry.

[5] Feigin A, Leenders KL, Moeller JR, Missimer J, Kuenig G, Spetsieris P (2001). Metabolic network abnormalities in early Huntington's disease: an [(18)F]FDG PET study. J Nucl Med.

[6] Mudali D, Teune LK, Renken RJ, Leenders KL, Roerdink JB (2015). Classification of parkinsonian syndromes from FDG-PET brain data using decision trees with SSM/PCA features. Comput Math Methods Med.

[7] Spetsieris PG, Ma Y, Dhawan V, Eidelberg D (2009). Differential diagnosis of parkinsonian syndromes using PCA-based functional imaging features. NeuroImage.

[8] Spetsieris PG, Eidelberg D (2011). Scaled subprofile modeling of resting state imaging data in Parkinson's disease: methodological issues. NeuroImage.

[9] Pagani M, Oberg J, De Carli F, Calvo A, Moglia C, Canosa A (2016). Metabolic spatial connectivity in amyotrophic lateral sclerosis as revealed by independent component analysis. Hum Brain Mapp.

[10] Vapnik VN (1995). The nature of statistical learning theory.

[11] Van Weehaeghe D, Ceccarini J, Delva A, Robberecht W, Van Damme P, Van Laere K (2016). Prospective validation of 18F-FDG brain PET discriminant analysis methods in the diagnosis of amyotrophic lateral sclerosis. J Nucl Med.

[12] Vandenberghe R, Nelissen N, Van Laere K, Thurfjell L, Buckley C, Farrar G, et al., editors. Support vector machine-based classification of 18F-flutemetamol scans in Alzheimer's disease, MCI and controls: comparison with visual reads and structural MRI-based classification. In: Alzheimer’s & Dementia: Elsevier; 2011. 10.1016/j.nicl.2015.10.007.

[13] Bicchi I, Emiliani C, Vescovi A, Martino S (2015). The big bluff of amyotrophic lateral sclerosis diagnosis: the role of neurodegenerative disease mimics. Neurodegener Dis.

[14] Gilbert RM, Fahn S, Mitsumoto H, Rowland LP (2010). Parkinsonism and motor neuron diseases: twenty-seven patients with diverse overlap syndromes. Mov Disord.

[15] Goldman JS, Quinzii C, Dunning-Broadbent J, Waters C, Mitsumoto H, Brannagan TH (2014). Multiple system atrophy and amyotrophic lateral sclerosis in a family with hexanucleotide repeat expansions in C9orf72. JAMA Neurol.

[16] Scholz SW, Bras J (2015). Genetics underlying atypical parkinsonism and related neurodegenerative disorders. Int J Mol Sci.

[17] Jucker M, Walker LC (2013). Self-propagation of pathogenic protein aggregates in neurodegenerative diseases. Nature.

[18] McCullagh PP, Nelder JA. Generalized linear models: Chapman and Hall; 1989. 10.1002/bimj.4710290217.

[19] Guyon I, Elisseeff A (2003). An introduction to variable and feature selection. J Mach Learn Res.

[20] Jolliffe IT (1986). Principal component analysis.

[21] Hastie T, Friedman J, Tibshirani R. Linear methods for classification. The elements of statistical learning: Springer series in Statistics; 2001. p. 79–113. 10.1007/978-0-387-84858-7.

[22] Zou H, Hastie T (2005). Regularization and variable selection via the elastic net. Journal of the Royal Statistical Society: Series B (Statistical Methodology).

[23] Varoquaux G, Kowalski M, Thirion B, editors. Social-sparsity brain decoders: faster spatial sparsity. International Workshop on Pattern Recognition in Neuroimaging (PRNI); 2016: IEEE.

[24] Cortes C, Networks VVS-V (1995). Mach Learn.

[25] Hofmann M (2006). Support vector machines—kernels and the kernel trick.

[26] Cristianini N, Shawe-Taylor J. An introduction to support vector machines and other kernel-based learning methods: Cambridge University Press; 2000. 10.1017/CBO9780511801389.

[27] Schrooten M, Smetcoren C, Robberecht W, Van Damme P (2011). Benefit of the Awaji diagnostic algorithm for amyotrophic lateral sclerosis: a prospective study. Ann Neurol.

[28] Friedman J, Hastie T, Tibshirani R (2010). Regularization paths for generalized linear models via coordinate descent. J Stat Softw.

[29] Robert P, Escoufier Y. A unifying tool for linear multivariate statistical methods: the RV- coefficient. Applied statistics, vol 3: WileyRoyal statistical society; 1976. 10.2307/2347233.

[30] Akdemir UO, Tokcaer AB, Karakus A, Kapucu LO (2014). Brain 18F-FDG PET imaging in the differential diagnosis of parkinsonism. Clin Nucl Med.

[31] Meyer PT, Frings L, Rucker G, Hellwig S (2017). (18)F-FDG PET in Parkinsonism: differential diagnosis and evaluation of cognitive impairment. J Nucl Med.

[32] Pagani M, Chio A, Valentini MC, Oberg J, Nobili F, Calvo A (2014). Functional pattern of brain FDG-PET in amyotrophic lateral sclerosis. Neurology.

[33] Baldassarre L, Mourao-Miranda J, Pontil M, editors. Structured sparsity models for brain decoding from fMRI data. In: Second International Workshop on Pattern Recognition in NeuroImaging: IEEE; 2012. 10.1109/PRNI.2012.31.

[34] Michel V, Gramfort A, Varoquaux G, Eger E, Thirion B (2011). Total variation regularization for fMRI-based prediction of behavior. IEEE Trans Med Imaging.

[35] van Weehaeghe D, Ceccarini J, Willekens SM, de Vocht J, van Damme P. Van Laere K. is there a glucose metabolic signature of spreading TDP-43 pathology in amyotrophic lateral sclerosis? Q J Nucl Med Mol Imaging. 2017. 10.23736/S1824-4785.17.03009-6. 10.23736/S1824-4785.17.03009-6 29166751

